# Nuclear cardiology practices and radiation exposure in Africa: results from the IAEA Nuclear Cardiology Protocols Study (INCAPS)

**DOI:** 10.5830/CVJA-2016-091

**Published:** 2017

**Authors:** Salah E Bouyoucef, Mathew Mercuri, Andrew J Einstein, Thomas NB Pascual, Ravi Kashyap, Maurizio Dondi, Diana Paez, Adel H Allam, Mboyo Vangu, João V Vitola, Nathan Better, Ganesan Karthikeyan, John J Mahmarian, Madan M Rehani, Andrew J Einstein

**Affiliations:** Centre Hospitalo-Universitaire de Bab El Ouéd, Alger, Algeria; Division of Cardiology, Department of Medicine, Columbia University Medical Center and New York-Presbyterian Hospital, New York, USA; Division of Cardiology, Department of Medicine, Columbia University Medical Center and New York-Presbyterian Hospital, New York, USA; Section of Nuclear Medicine and Diagnostic Imaging, Division of Human Health, International Atomic Energy Agency, Vienna, Austria; Section of Nuclear Medicine and Diagnostic Imaging, Division of Human Health, International Atomic Energy Agency, Vienna, Austria; Section of Nuclear Medicine and Diagnostic Imaging, Division of Human Health, International Atomic Energy Agency, Vienna, Austria; Section of Nuclear Medicine and Diagnostic Imaging, Division of Human Health, International Atomic Energy Agency, Vienna, Austria; Cardiology Department, Al Azhar University, Cairo, Egypt; Department of Nuclear Medicine, Charlotte Maxeke Johannesburg Academic Hospital, University of the Witwatersrand, Johannesburg, South Africa; Quanta Diagnóstico & Terapia, Curitiba, Brazil; Department of Nuclear Medicine, Royal Melbourne Hospital and University of Melbourne, Melbourne, Australia; Department of Cardiology, All India Institute of Medical Sciences, New Delhi, India; Department of Cardiology, Houston Methodist DeBakey Heart and Vascular Center, Houston, Texas, USA; Radiation Protection of Patients Unit, International Atomic Energy Agency, Vienna, Austria; and Department of Radiology, Massachusetts General Hospital, Harvard Medical School, Boston, Massachusetts, USA; Department of Radiology, Columbia University Medical Center and New York-Presbyterian Hospital, New York, USA

**Keywords:** myocardial perfusion imaging, radiation, effective dose, best practices, Africa

## Abstract

**Objective:**

While nuclear myocardial perfusion imaging (MPI) offers many benefits to patients with known or suspected cardiovascular disease, concerns exist regarding radiationassociated health effects. Little is known regarding MPI practice in Africa. We sought to characterise radiation doses and the use of MPI best practices that could minimise radiation in African nuclear cardiology laboratories, and compare these to practice worldwide.

**Methods:**

Demographics and clinical characteristics were collected for a consecutive sample of 348 patients from 12 laboratories in six African countries over a one-week period from March to April 2013. Radiation effective dose (ED) was estimated for each patient. A quality index (QI) enumerating adherence to eight best practices, identified a priori by an IAEA expert panel, was calculated for each laboratory. We compared these metrics with those from 7 563 patients from 296 laboratories outside Africa.

**Results:**

to that of the rest of the world [9.1 (5.1–15.6) vs 10.3 mSv (6.8–12.6), p = 0.14], although a larger proportion of African patients received a low ED, ≤ 9 mSv targeted in societal recommendations (49.7 vs 38.2%, p < 0.001). Bestpractice adherence was higher among African laboratories (QI score: 6.3 ± 1.2 vs 5.4 ± 1.3, p = 0.013). However, median ED varied significantly among African laboratories (range: 2.0–16.3 mSv; p < 0.0001) and QI range was 4–8.

**Conclusion:**

Patient radiation dose from MPI in Africa was similar to that in the rest of the world, and adherence to best practices was relatively high in African laboratories. Nevertheless there remain opportunities to further reduce radiation exposure to African patients from MPI.

## Objective:

The increasing burden of cardiovascular disease, affecting rates of morbidity and mortality, has brought with it a rise in technological innovations for diagnosing and managing disease. In recent decades, cardiovascular imaging modalities that use ionising radiation have become essential to cardiology practice. Myocardial perfusion imaging (MPI) is one such technology. Through the use of an injected radiopharmaceutical in conjunction with a single-photon emission computed tomography (SPECT) or positron emission tomography (PET)camera, MPI provides information that can be used to both diagnose coronary artery disease and stratify risk, and thus guide patient management.^1^-^3^

While the benefits of MPI are apparent, there are concerns regarding potential harmful effects from radiation exposure as a result of undergoing this procedure.^4^ Therefore, organisations promoting radiation safety, such as the International Commission on Radiological Protection (ICRP), advocate practice to use radiation only when justified and to limit radiation to levels that are ‘as low as reasonably achievable’ (ALARA) without compromising on diagnostic information.^5^

While judicious application of imaging technology that employs ionising radiation is the best approach, numerous techniques or ‘best practices’ exist to assist practitioners in limiting the dose when its use is warranted.^6^-^9^ A recent study conducted by the International Atomic Energy Agency (IAEA) revealed significant variation in both the uptake of evidencebased best practices to reduce patient radiation exposure and patient radiation effective dose from MPI among nuclear cardiology laboratories worldwide.^9^

Beyond this study, little is known about nuclear cardiology practice with regard to radiation exposure around the world, especially among laboratories in Africa. The purpose of this study was to characterise the MPI practice and patient dose among African nuclear cardiology laboratories participating in the IAEA Nuclear Cardiology Protocols Study (INCAPS), and examine it relative to practice among laboratories worldwide.

## Methods

This study used patient and laboratory data collected as part of the worldwide INCAPS survey. INCAPS was initiated by an expert committee of physicians and medical physicists convened by the IAEA to characterise radiation doses and examine best-practice use to minimise dose in nuclear cardiology clinics around the world.

INCAPS used a cross-sectional design whereby participating laboratories provided demographics, clinical characteristics and MPI study parameters for a consecutive sample of patients over a one-week period of the laboratory’s choice between 18 March and 22 April 2013. Nuclear cardiology laboratories were recruited through membership lists provided by a number of national and international cardiology or nuclear medicine societies. A designated local investigator prospectively acquired data using a standardised data-collection tool that was issued by the coordinating centre. This included information on the patient’s age, gender and weight, and MPI study parameters, such as injected radiopharmaceutical, administered activity, camera type, imaging position and protocol, and camera-based dose-mediating hardware or software. Full details regarding study design are presented elsewhere.^9^

The study was reviewed, approved and conducted in compliance with the Columbia University institutional review board, which deemed it exempt from the requirements of US federal regulations for the protection of human subjects (45 CFR 46) because no individually identifiable health information was collected.

Radiation dose was calculated for each patient using the effective dose (ED), as per the dose coefficients provided by the ICRP.^10^ ED is a whole-body measure that reflects both estimated individual organ doses and their relative sensitivity to carcinogenic effects from radiation. Radiation dose from studies using rubidium-82 was calculated using Senthamizhchelvan’s conversion coefficients.^11^ All radiation doses are presented in units of millisieverts (mSv). Mean and median ED was calculated at the laboratory and regional (Africa vs rest of the world) level.

Using current clinical practice guidelines, the expert committee convened by the IAEA identified eight ‘best practices’ for optimising radiation dose from MPI studies a priori. A laboratory’s adherence to each of these practices was evaluated from the acquired data. Details regarding how the best practices are defined and adherence scored were reported previously by Einstein et al.^9^ and are summarised in Table 1.

**Table 1:** Scoring and explanations of the eight best practices. Adapted from Einstein et al.^9^

*Best practice*	*Scoring*	*Explanation*
Avoid thallium stress	One point if no thallium-201 studies were performed in patients ≤ 70 years old	SPECT imaging with thallium is associated with a considerably higher radiation dose to patients compared with technetium-based radiopharmaceuticals. This item excludes thallium viability studies and stress redistribution–re-injection stress and viability studies
Avoid dual isotope	One point if no dual isotope (rest thallium and stress technetium) studies were performed in patients ≤ 70 years old	Dual isotope imaging is associated with the highest radiation dose of any protocol
Avoid too much technetium	One point if (1) no study was performed with technetium activities > 1 332 MBq (36 mCi), and (2) mean total effective dose was < 15 mSv for all studies with two technetium injections	1 332 MBq is the highest recommended activity in guidelines, and 15 mSv is a very high radiation dose for a 99mTc study
Avoid too much thallium	One point if for each study with thallium, less than 129.5 MBq was administered at stress	The expert committee maintained that 129.5 MBq should be the upper threshold for thallium activity
Perform stressonly imaging	One point if the laboratory performed at least one stress-only study, in which rest imaging was omitted, or if the laboratory did only PET-based stress tests	If stress images are completely normal, subsequent rest imaging can be omitted
Use camerabased dosereduction strategies	One point if the laboratory performed at least one study using at least one of the following: (1) attenuation correction (CT or transmission source), (2) imaging patients in multiple positions, e.g. both supine and prone, (3) high-technology software (e.g. resolution recovery and noise reduction), and (4) high-technology hardware (e.g. PET or a solid-state CZT SPECT camera)	Each of these approaches reduces the administered activity needed and facilitates performance of stress-only imaging
Weight-based dosing for technetium	One point if the laboratory had a statistically significant positive correlation between patient weight and administered activity (MBq), for injections of technetium	Tailoring the administered activity to the patient weight offers an opportunity to reduce radiation dose
Avoid inappropriate dosing that can lead to ‘shine-through’ artifact	One point if the laboratory performed no SPECT studies with technetium rest and stress injections on the same day, in which the activity of the second injection was less than three times that of the first injection	Shine through occurs in one-day technetium studies when residual radioactivity from the first injection interferes with the images for the second injection. To avoid shine through, guidelines recommend that the activity for the second injection should be three to four times higher than the first injection. A second injection of less than three times of the activity of the first injection constituted a dosing that can lead to shine through

Briefly, these best practices centred around the practice of avoiding administering higher-than-needed doses of radiopharmaceuticals, using a strategy of stress-only imagingwhere possible, avoiding dosing leading to ‘shine through’artifact, and the use of camera-based dose-reduction softwareor hardware technology (e.g. resolution recovery software ortwo-position supine and prone imaging), which can reduce radiation dose. Stress-only imaging refers to a protocol whereby rest imaging is only acquired in the event that stress images, which are performed first, reveal abnormalities. The use of this protocol has been shown to reduce radiation exposure, as a significant proportion of the population will not require the subsequent rest imaging.^12^ When performing single-day, two-injection technetium studies, there is a possibility of residual activity from the first injection interfering with the interpretation of images from the second injection. This shine-through artifact can be avoided by ensuring the administered activity in the second injection is more than three times that of the first injection.

A composite quality index (QI) was enumerated for each laboratory, based on the number of specified best practices followed during the observation period. The expert committee established a median laboratory ED of ≤ 9 mSv, as specified in professional society recommendations,^13^ and a QI score of ≥ 6 as benchmarks for desirable laboratory performance.

## Statistical analysis

The primary comparison examined patient ED and laboratory best-practice adherence differences between Africa and the rest of the world. As a second focus, ED was compared between laboratories within Africa. For continuous variables, normality was tested using the Kolmogorov–Smirnov test for patient-level comparisons, given the large sample size of 7 911, and using the Shapiro–Wilk test for laboratory-level comparisons. Continuous variables were compared in terms of means using the Student’s t-test or analysis of variance for normally distributed data and compared in terms of medians using the Kruskal–Wallis test for non-normally distributed data. The chi-squared test was used to compare categorical variables. All analyses were performed using Stata/SE 13.1 (StataCorp, College Station, TX) and a p-value < 0.05 was considered statistically significant.

## Results

Of the 30 laboratories performing MPI in Africa, identified in the IAEA Nuclear Medicine database, data collection yielded information on 348 consecutive MPI studies from 12 laboratories in Algeria, Egypt, Kenya, Senegal, South Africa and Tunisia. It was compared with 7 563 studies from 296 laboratories in 59 countries around the world.

Patient demographics, clinical characteristics and radiation dose information for the African and non-African study cohorts are presented in Table 2. African patients were younger compared to patients from the rest of the world (60.2 ± 11.0 vs 64.3 ± 12.0 years; p < 0.0001). Median and mean patient ED were similar in both populations. However, a larger proportion of African patients received an ED ≤ 9 mSv (49.7 vs 38.2%, p < 0.001).

**Table 2:** Patient and laboratory demographics and clinical characteristics

*Patients*	*Africa (n = 348)*	*Rest of world (n = 7 563)*	*p-value*
Female, n (%)	135 (38.8)	3119 (41.2)	0.36
Age (years)			
Mean	60.2	64.3	< 0.0001
SD	11	12	
Effective dose (mSv)			
Median	9.1	10.3	0.14
IQR	5.1–15.6	6.8–12.6	
Range	1.8–20.0	0.75–35.6	
≤ 9 mSv, n (%)	173 (49.7)	2892 (38.2)	< 0.001
Stress-only, n (%)	109 (31.3)	896 (11.8)	< 0.001
PET, n (%)	6 (1.7)	465 (6.1)	< 0.001
Laboratories	12	296	
Patients/laboratories			
Median	19	16	0.402
IQR	10–48	8–33	
Range	4–73	1–250	
Quality index score			
≥ 6, n (%)	9 (75.0)	133 (44.9)	0.041
Mean	6.3	5.4	0.013
SD	1.2	1.3	
Laboratories with median dose ≤ 9 mSv, n (%)	5 (41.7)	86 (29.1)	0.35

SD, standard deviation; IQR, interquartile range

**Fig. 1.**
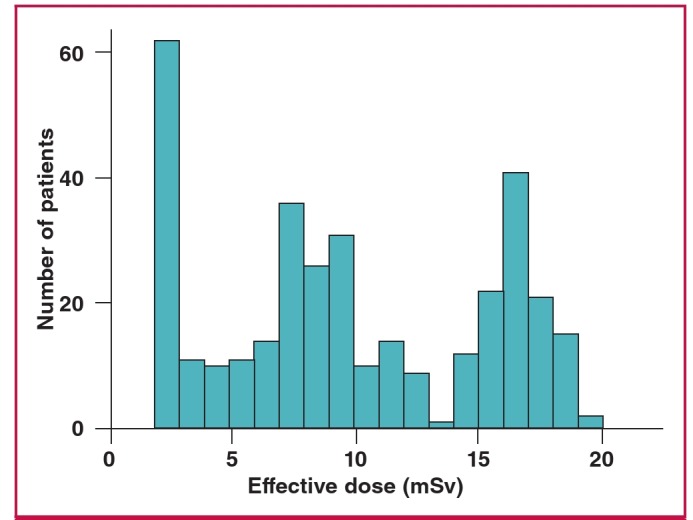
Distribution of radiation-effective dose among observed African patients.

The distribution of individual African patient EDs is presented in Fig. 1. African patients were more likely than non-Africans to undergo an MPI study using a stress-only protocol [odds ratio (OR): 3.4, 95% CI: 2.7–4.3, p < 0.001]. The use of PET imaging was lower in African patients (1.7 vs 6.1%, p < 0.0001).

Patient volumes were similar for participating African and non-African laboratories, as were the laboratory mean and median ED and the proportion of laboratories with a median ED ≤ 9 mSv (Table 2). However, there was significant variation in the ED among African laboratories (median ED range: 2–16.3 mSv, p < 0.0001). African laboratory volumes and EDs are presented in Table 3.

**Table 3:** African laboratory patient volume, radiation exposure and quality index score

**	*No of patients*	*Effective dose (mSv)*			*Quality index*
*25%*		*Median*	*75%*	*(QI) score*
Algeria 1	42	5	8.4	9.5	7
Algeria 2	73	1.8	2	6.2	7
Algeria 3	17	2.5	4.5	5.1	7
Algeria 4	14	11.3	11.9	12.5	6
Egypt 1	54	8.2	16.3	17.2	8
Egypt 2	39	7.3	15.3	16	8
Kenya 1	5	11.4	11.6	11.8	4
Senegal 1	4	6.3	8	8.9	5
South Africa 1	12	9.4	9.4	9.4	5
South Africa 2	60	14.8	16.1	17.8	6
Tunisia 1	21	8.2	9.4	9.9	7
Tunisia 2	7	3.1	3.6	9	6

The overall adherence to best practices to minimise radiation exposure was higher among African laboratories, as reflected in the mean QI score (6.3 ± 1.2 vs. 5.4 ± 1.3, p = 0.013) and the proportion of laboratories with a QI score ≥ 6 (75.0 vs 44.9%, p = 0.041). However, while the adherence to each individual best practice was also higher among African laboratories, this difference failed to reach statistical significance (Table 4). The only exception was the use of stress-only imaging, which was used in 66.7% of African laboratories and only 28.7% of non-African laboratories (p = 0.005). The practices of weightbased dosing and ensuring sufficient administered activity to avoid shine through were both higher in African laboratories but failed to reach statistical significance.

**Table 4:** Laboratory best-practice adherence

*Best practice*	*Africa (n = 12)*		*Rest of world (n = 296)*		*p-value*
	*n*	*%*	*n*	*%*	**
Avoid thallium stress	12	100.0	270	91.2	0.61
Avoid dual isotope	12	100.0	286	96.6	1
Avoid too much technetium	11	91.7	252	85.1	1
Avoid too much thallium	12	100.0	294	99.3	1
Perform stress-only imaging	8	66.7	85	28.7	0.005
Use camera-based dosereduction strategies	8	66.7	198	66.9	1
Weight-based dosing for technetium	6	50.0	82	27.7	0.108
Avoid shine through	7	58.3	129	43.6	0.313

## Discussion

Africa is facing difficulties in developing nuclear medicine in general,^14^ and the continent has the lowest ratio of clinical nuclear medicine applications per capita. Few studies^15^,^16^ explicitly examine nuclear cardiology practice in Africa. The INCAPS worldwide cross-sectional study of MPI provides a valuable opportunity to better appreciate radiation doses and use of best practices to reduce radiation among patients undergoing nuclear cardiology procedures on the African continent, and how Africa compares to the rest of the world in terms of patient dose and best-practice adherence.

Analysis of data from INCAPS revealed that overall radiation dose to patients undergoing a procedure in Africa was similar to that among patients undergoing a procedure elsewhere in the world. Notably, African laboratories performed much better than the rest of the world with regard to best-practice adherence to minimise patient dose, as reflected in both a higher QI score and proportion of laboratories adhering to each best practice. However significant variation in ED and QI score was noted within Africa, specifically at the laboratory level.

The eight-fold range in median patient ED at the laboratory level is likely attributable to protocol use, specifically the practice of stress-only imaging. While this practice was used in the majority of African laboratories, the rate of use was higher in some than others. One laboratory in particular used a stress-only protocol in 73% of its cases and had the lowest median ED, and incidentally, the highest patient volume.

The option of stress-only protocol could be a consequence of a few factors: the desire to lower radiation dose, the overload of patients due to insufficient nuclear cardiology facilities inducing a long waiting list, and/or for economic reasons to reduce the cost of MPI. But regardless of the laboratory’s motivation to commonly use a stress-only protocol, its salutary effect on radiation dose is undeniable. By contrast, the laboratory with the highest mean ED used a stress-only protocol in only 1.7% of cases.

However while a practice of stress-only imaging is desirable where indicated, the correct rate is determined by the disease rate in the imaged population. Therefore it is difficult to determine the extent to which the observed rates of stress-only use reflect over- or under-use of this protocol, or of MPI imaging more generally.

Overall there was good adherence in the use of specified best practices among the observed African laboratories. However, in contrast with the results of the worldwide study, there is a seemingly poor correlation between laboratory adherence to best practices and mean patient ED in Africa. Surprisingly, the two laboratories that adhered to all eight best practices were among the laboratories with the highest patient ED on the continent. These laboratories predominantly used two-day protocols, with rest imaging performed on the second day, a fact that might explain the relatively higher rates of stress-only use in these laboratories (29.6 and 38.5%), compared to other observed laboratories in INCAPS. This suggests that dose-minimisation strategies are not strictly limited to the best practices described.

Likewise, adherence to a best practice as we have defined it does not mean it is optimally applied in the laboratory. For example, a laboratory might use a high-efficiency solidstate SPECT camera for every case, but may never use prone positioning (both camera-based dose-reduction strategies).

Laboratories should continue to be vigilant in ensuring that patient doses are optimised, given the high doses involved in theMPI study. Still, the lack of complete adherence to best practices among the majority of laboratories suggests opportunity tofurther reduce patient EDs through those practices specified by the expert committee. Furthermore this should be possible even in environments with significant resource constraints, as all of the specified best practices can be implemented with no additional cost, and in some cases, a cost reduction.

Changing demographics and increased socio-economic development on the African continent are contributing to a rise in chronic illness, especially cardiovascular disease.^17^,^18^ Commensurate with this rise will be increased demand for diagnostic imaging. Resource constraints and a lack of available expertise have been cited as challenges to providing nuclear cardiology procedures in Africa.^15^,^19^ Furthermore, few nuclear cardiology-capable centres exist in Africa, even compared to developing nations else where in the world; their equipment is older, and practitioners perceive a high need for additional training in a variety of nuclear medicine techniques.^16^

The data presented here may reflect these challenges. For example, the use of PET imaging among observed African laboratories was quite low, likely owing to lack of access to the relatively expensive scanners and/or radiopharmaceuticals, which in many cases require on-site manufacture. Likewise, the high use of stress-only imaging may be a result of careful use of scarce radiopharmaceuticals and camera time on the part of African nuclear medicine physicians. On the other hand, the lack of nuclear cardiology infrastructure (and therefore opportunity for training physicians and developing expertise) relative to other regions in the world may have contributed to the development of regional centres of excellence.^20^

Despite the increasing prevalence of coronary artery disease in Africa, which has accompanied westernisation, owing to limitations in trained personnel and equipment, nuclearcardiology capabilities presently exist in few African countries.Even in those countries where there are MPI capabilities, thereare few laboratories. These are concentrated in regional referralcentres, many of which are university-based teaching hospitalsreceiving technical assistance from IAEA. This assistance is provided through the IAEA’s technical cooperation programme.

Under this programme, every year regional training courses are organised on specific nuclear medicine topics, with a major focus on nuclear cardiology. Participants from all over the African region, financially supported by the technical cooperationprogramme, gather in the host centres to attend lectures andpractical sessions given by international experts. Furthermore, the IAEA provides financial support to fellowships that maylast two to three months or up to years, to train future leadersin the field and develop regional centres of excellence. For manycentres, the programme also supports the purchase of equipmentand even SPECT cameras. The high rate of adherence to bestpractices observed among African laboratories is consistent with the concept of centres of excellence.

## Limitations

Our study has some limitations. There is no comprehensive list of laboratories performing nuclear cardiology procedures in many countries, therefore it is not clear to what extent data acquired in the INCAPS study represents typical MPI practicearound the world. Furthermore, the low number of participating laboratories in Africa may further exacerbate this concern. One could argue that the participation rate in Africa reflects the relative lack of nuclear cardiology-capable laboratories on the continent – such laboratories are known by the IAEA to exist in only eight countries, six of which are represented in this study. It is possible that those laboratories that did participate are more engaged with the international radiation protection community, and therefore patient ED and best-practice adherence data presented here may represent the best-case scenario among African laboratories. Unfortunately we could not determine the response rate for participation, as the multiple mechanisms used to contact laboratories contain some overlap. However our study did manage to recruit 12 of 30 (40%) of the laboratories performing MPI in Africa, identified by the IAEA database.

In addition, this study did not assess image quality or patient outcomes. Therefore we cannot determine whether lower patient ED and high uptake to the specified best practices indeed translate to improved patient care. Finally, the fact that two laboratories that adhered to all eight best practices showed overall higher ED suggests that our metrics may not be sensitive to the African MPI environment. Future research should examine how to optimise dose-reduction best practices to the local context.

## Conclusion

Our study of nuclear cardiology practice reveals that African laboratories performed better than the rest of the world withregard to best-practice adherence to optimise patient radiationdose. However wide variation in practice still exists and greateruptake of stress-only imaging, use of camera-based dosereductiontechnologies, and optimised dosing protocols may provide additional opportunity to further reduce radiation exposure from MPI in Africa, often at no extra cost to care.
